# Spatial distribution and temporal trends of AIDS in Brazil and regions between 2005 and 2020

**DOI:** 10.1590/1980-549720230002

**Published:** 2023-01-06

**Authors:** Jefferson Felipe Calazans Batista, Marília Ramalho Oliveira, Débora Lorena Melo Pereira, Maria Laura Sales da Silva Matos, Isabela Teles de Souza, Max Oliveira Menezes

**Affiliations:** I Universidade Tiradentes – Aracaju (SE), Brazil.; II Universidade Estadual do Maranhão – Caxias (MA), Brazil.; III Universidade Federal do Maranhão – São Luis (MA), Brazil.

**Keywords:** Time series studies, AIDS, Spatial analysis, Epidemiology, Incidence, Estudos de séries temporais, AIDS, Análise espacial, Epidemiologia, Incidência

## Abstract

**Objective:**

To analyze the spatial distribution and the temporal trend of the AIDS incidence rate in Brazil from 2005 to 2020.

**Methods:**

This is an ecological, temporal, and spatial study on AIDS cases in Brazil. Data from the Notifiable Diseases Information System were stratified by year of diagnosis, region of the country/municipalities of residence, and age group (over 13 years). Incidence rates were calculated for temporal estimation using the Joinpoint model, as well as Spatial Empirical Bayes (SEB) for spatial distribution, using the Kernel density estimator.

**Results:**

The incidence rate in Brazil, in 2020, was 17.69 cases per 100 thousand inhabitants. The general trend (2005–2020) was decrease in Brazil (Annual Percent Change – APC=-2.0%), in the Southeast (APC=-4.4%) and South (APC=-3.0%) regions. The North (APC=2.3%) showed an increase trend, whereas the Southeast and Midwest regions were stationary (p>0.05). Brazil, Southeast, South, and Midwest regions showed a decrease trend in most age groups. The Northeast and North regions showed an increase in the age groups of 13–29 years and 13–24 years, respectively. The Kernel estimator showed clusters with SEB above 30/10 thousand inhabitants in the states of Paraíba, Sergipe, Alagoas, Pernambuco, São Paulo, Minas Gerais, Pará, Rio Grande do Sul, and Santa Catarina.

**Conclusion:**

Brazil, the Southeast, and South regions showed a decrease in the incidence rate, whereas the North region increased and the Northeast and Midwest regions were stationary. The Southeast, South, and Northeast regions presented the largest clusters of SEB.

## RESUMO

**Objetivo:**

Analisar a distribuição espacial e a tendência temporal da taxa de incidência de AIDS no Brasil no período de 2005 a 2020.

**Métodos:**

Estudo ecológico, temporal e espacial sobre os casos de AIDS no Brasil. Dados provenientes do Sistema de Informação de Agravos de Notificação do Departamento de Informática do Sistema Único de Saúde foram estratificados por ano do diagnóstico, região do país/municípios de residência e faixa etária (acima de 13 anos). Foram calculadas as taxas de incidência (TI) para a estimativa temporal por meio do modelo de *joinpoint*, bem como as taxas bayesianas empíricas espaciais (TBEE) para a distribuição espacial pelo estimador de densidade de Kernel.

**Resultados:**

A TI no Brasil no ano de 2020 foi de 17,69 casos para cada 100 mil habitantes. A tendência geral (2005–2020) foi de diminuição no Brasil (variação percentual anual — VPA=-2,0%), Sudeste (VPA=-4,4%) e Sul (VPA=-3,0%). O Norte (VPA=2,3%) demonstrou aumento, enquanto o Sudeste e Centro-oeste foram estacionários (p>0,05). O Brasil, Sudeste, Sul e Centro-oeste apresentaram tendência de diminuição na maioria das faixas etárias. O Nordeste e Norte apresentaram aumento nas faixas etárias de 13 a 29 anos e 13 a 24 anos, respectivamente. O estimador de Kernel demonstrou conglomerados com TBEE acima de 30/10 mil habitantes nos estados de Paraíba, Sergipe, Alagoas, Pernambuco, São Paulo, Minas Gerais, Pará, Rio Grande do Sul e Santa Catarina.

**Conclusão:**

O Brasil e as Regiões Sudeste e Sul apresentaram diminuição da TI, enquanto o Norte aumentou e o Nordeste e Centro-oeste foram estacionários. As Regiões Sudeste, Sul e Nordeste apresentaram os maiores conglomerados das TBEE.

## INTRODUCTION

Human immunodeficiency virus (HIV) infection and its acquired immunodeficiency syndrome (AIDS) are considered a serious global health issue. Many scholars classify them as an epidemic; others, as a pandemic, due to their presence in every region of the world^1,2^.

The global burden of people living with AIDS in 2019 was 36.9 million, which corresponds to 0.5% of the world population, with a prevalence of 476 cases per 100 thousand inhabitants. In addition, infection rates tend to increase in several regions such as North America, South America, Oceania, and Europe. It should be noted that these rates overlap with population growth despite prevention and treatment strategies^3^.

In Brazil, in 2020, approximately 29 thousand cases of AIDS were diagnosed, including 10,417 deaths due to this cause^4^. The Brazilian epidemic profile follows a global pattern, in which the prevalence is higher for specific population groups such as gays, bisexuals, homosexuals, and transgender women^5^.

In this context, international agreements support the reduction in the number of AIDS, with projections for 2030. Thus, since 2016, the 90-90-90 global target has been developed, namely: 90% of people living with AIDS should know their HIV status; 90% of those diagnosed should be receiving treatment; and 90% of people undergoing treatment should have suppressed viral loads^6^. However, these goals were not achieved, as in 2019 only 81% of people living with the virus knew their HIV status; just over 60% of individuals had access to treatment; and 59% individuals became “undetectable”^7^.

This type of data demonstrate the critical context of AIDS as well as the difficulty in achieving the goals proposed by the United Nations (UN) for 2030. The multifactorial aspects that contribute to differences in the distribution of the virus/disease in the population at both social and economic levels are noteworthy. The risk of infection and access to treatment, for instance, are affected by homophobia, racism, social inequalities, and lack of services aimed at vulnerable groups. In underdeveloped countries, even more precarious situations are observed due to the lack of testing and treatment for infected people, which consequently increases the incidence, prevalence, and mortality from the disease^8^.

The increase in the incidence of AIDS cases is an alarming finding, especially within the Brazilian context, which has had public investments for decades to prevent and mitigate damages related to this infection. In addition, Brazil has a large database for health surveillance, a fact that allows us to understand the behavior of the condition from a spatial and temporal perspective. Hence, conducting research that promotes the continuous discussion on this condition is essential, thus supporting the updating and expansion of public health policies. In this study we aim to analyze the spatial distribution and the temporal trend of the AIDS incidence rate in Brazil from 2005 to 2020.

## METHODS

This is an ecological study with spatial and temporal analysis, with a quantitative approach, descriptive and exploratory in nature, which used data on confirmed AIDS cases in Brazil and its regions from 2005 to 2020. Data were collected from the Notifiable Diseases Information System (*Sistema de Informação de Agravos de Notificação* – SINAN), available from the Information Technology Department of the Brazilian Unified Health System (DATASUS), accessed via the TABNET tabulator.

For the present study, a temporal analysis from 2005 onwards was considered, as the data referring to AIDS cases from SINAN alone were aggregated into the cases of the Mortality Information System (*Sistema de Informação sobre Mortalidade* – SIM) and those from the Control System for Laboratory Tests of the National CD4+/CD8+ Lymphocyte Count and HIV Viral Load Network (*Sistema de Controle de Exames Laboratoriais de CD4+/CD8+ e Carga Viral do HIV* – SISCEL) in 2004^9^.

The variables selected for analysis were: year of diagnosis (from 2005 to 2020), region of residence in Brazil (North, Northeast, Southeast, South, Midwest, and Brazil), and age group over 13 years, as it characterizes an adult AIDS case^10^.

The AIDS incidence rate (IR) was estimated by region of the country and age group, using the following formula:


$$IR=\;\frac{N\;of\;AIDS\;in\;a\;given\;place,\;period\;and\;age\;group}{population\;residing\;in\;the\;same\;place,\;period\;and\;age\;group}\times 100,000$$

The intercensal estimates from the Brazilian Institute of Geography and Statistics (IBGE), from 2005 to 2020, were used as population base for the calculation^11^.

To estimate the trend, the Joinpoint^12^ regression model was adopted, which allows analyzing temporal trends (incidence rate, mortality, survival, or prevalence). The test is based on the Monte Carlo permutation method, in which several trend models are estimated and the one that best represents the observed pattern is chosen^13^.

Thus, for using the model, AIDS incidence rates (according to age group and region of the country) were considered as the dependent variable; and the years, as the independent variable. The model was adjusted by logarithmic transformation of the dependent variable {In(y)=xb}; the standard error of the incidence rate, calculated according to indications in the literature^14^; and by correcting the first-order autocorrelation estimated from the data. The results are presented as annual percent change (APC). For the present study, APC values throughout the analysis period (2005–2020) were considered. Positive or negative values, when statistically significant (p<0.05), indicate increase and decrease trends, respectively, while non-significant values indicate a stationary pattern^13^.

For spatial analysis, the Kernel density estimator was adopted. This estimate is a set of nonparametric statistical procedures that generate a density surface by smoothing points, forming “hot spots” that indicate clusters in a geographical distribution^15^. The points were produced from the centroids of the municipalities. Spatial Empirical Bayes (SEB) were used to draw the map. These rates are intended to reduce the variability of the estimates, restricting random fluctuation. According to Carvalho et al.^16^, the SEB is more appropriate for maps with large regional differences or many polygons, as it considers the neighborhood matrix (local average).

The cartographic base (territorial meshes) was provided by IBGE in the 2020 version. The projection corresponded to the Universal Transverse Mercator (UTM) coordinate system, using the Geocentric Reference System for the Americas 2000 model.

The Joinpoint Regression Program version 4.8.0.1 (Surveillance Research Program, 2022) was adopted for trend calculations The Kernel estimator was performed using the QGIS 4.24 Tisler software^17^. To generate the SEB, the GeoDA 1.20 software was used. The confidence interval (CI) of 95% and the significance level of 5% (p<0.05) were adopted for all estimates.

This study is exempt from assessment of the Research Ethics Committee because it has, as a source of information, publicly accessible secondary data that do not address information at an individual level. However, all the precepts and guidelines presented in Resolution No. 510, of 2016, of the National Commission for Research Ethics (CONEP)^18^ were respected.

## RESULTS

Over the 16 years of the present study, Brazil recorded 623,158 cases of AIDS. The Southeast region accounted for 42.7% (n=266,086) of the total, followed by the South, with 20.9% (n=130,533); the Northeast, with 20.2% (n=126,189); the North, with 9.2% (n=57,384); and the Midwest, with 69% (n=42,966).

**Figure 1:**
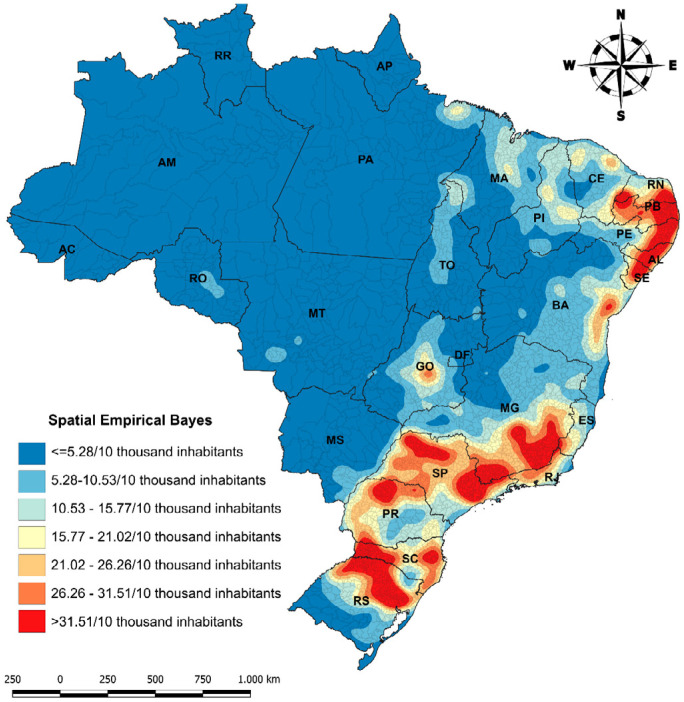
Kernel density map of Spatial Empirical Bayes of AIDS in the period from 2005 to 2020.

Source: Research data, 2022.

The highest AIDS incidence rates were recorded in the South, followed by the North, Brazil, Southeast, Midwest, and Northeast (Table 1, Figure 1 Supplementary Material).

**Table 1:** Number of cases and incidence rate of AIDS in Brazil and its regions from 2005 to 2020.

Year	North		Northeast		Southeast		South		Midwest		Brazil	
	n	Rate*	n	Rate*	n	Rate*	n	Rate*	n	Rate*	n	Rate*
2005	1,989	20.83	5,872	16.31	19,405	33.04	7,466	37.34	2,246	23.86	36,978	27.66
2006	2,115	21.55	5,644	15.40	18,516	31.03	8,135	40.02	2,217	22.97	36,627	26.91
2007	2,438	24.18	6,461	17.34	17,608	29.06	8,883	43.00	2,335	23.61	37,725	27.24
2008	3,004	29.01	7,000	18.48	18,129	29.47	9,691	46.17	2,469	24.36	40,293	28.60
2009	3,109	29.26	7,355	19.12	18,459	29.56	8,866	41.59	2,547	24.54	40,336	28.16
2010	3,337	30.61	7,594	19.45	17,792	28.08	8,694	40.17	2,585	24.32	40,002	27.48
2011	3,359	30.06	8,036	20.29	18,498	28.78	9,386	42.73	2,833	26.04	42,112	28.48
2012	3,459	30.21	8,542	21.26	17,698	27.16	9,165	41.13	3,003	26.99	41,867	27.87
2013	4,317	36.83	9,035	22.18	17,271	26.14	9,159	40.53	3,057	26.89	42,839	28.09
2014	4,462	37.20	8,825	21.36	16,765	25.03	8,642	37.74	2,928	25.22	41,622	26.89
2015	4,296	35.04	8,900	21.25	16,157	23.81	8,449	36.44	2,788	23.53	40,590	25.85
2016	4,424	35.28	8,766	20.65	15,548	22.62	7,621	32.46	2,715	22.45	39,074	24.53
2017	4,141	32.31	9,016	20.96	14,989	21.54	7,246	30.49	2,859	23.18	38,251	23.68
2018	4,544	34.71	9,152	21.00	14,337	20.37	6,959	28.95	2,845	22.63	37,837	23.12
2019	4,786	35.82	8,957	20.30	13,586	19.09	6,883	28.32	3,115	24.33	37,327	22.52
2020	3,604	26.44	7,034	15.76	11,328	15.75	5,288	21.53	2,424	18.60	29,678	17.69

* Incidence rate of AIDS per 100 thousand inhabitants.

Source: Research data, 2022.

The temporal trend in the incidence rate of AIDS in Brazil was decrease in the general group and in the 30–34, 35–39, 40–49, and 50–59 age groups. The Southeast and South regions were the only regions with a predominance of decrease in the trend; the first showed this pattern in almost all age groups. In the Northern region, we found a general increase trend, and we verified this increase pattern also in the younger age groups (up to 25–29 years). In the Northeast, the general trend was stationary, with an increase in the 13–9, 20–24, 50–59, and 60+ age groups; in addition, we verified decrease only the 35–39 age group. The Midwest showed increase in the 20–24 age group and a decrease in the 30–34, 35–39, and 40–49 age groups (Table 2).

**Table 2:** Temporal trend of the AIDS incidence rate, according to age group, in Brazil and its regions from 2005 to 2020.

Age group		APC (%)	95%CI	Interpretation	Age group	APC (%)	95%CI	Interpretation
**Brazil**					**Southeast**			
General		-2.0*	-2.7; -1.2	Decrease	General	-4.4*	-5.8; -2.9	Decrease
	13–19	0.5	-0.3; 1.4	Stationary	13–19	-1.1*	-1.8; -0.3	Decrease
	20–24	1.8	-0.3; 4	Stationary	20–24	0.5	-1.7; 2.8	Stationary
	25–29	-1	-2.4; 0.3	Stationary	25–29	-2.4*	-3.1; -1.6	Decrease
	30–34	-3.3*	-4; -2.6	Decrease	30–34	-5.0*	-6.6; -3.3	Decrease
	35–39	-4.1*	-4.8; -3.4	Decrease	35–39	-6.5*	-8.4; -4.6	Decrease
	40–49	-3.1*	-3.9; -2.3	Decrease	40–49	-5.3*	-5.9; -4.8	Decrease
	50–59	-1.4*	-2.4; -0.4	Decrease	50–59	-4.0*	-4.9; -3	Decrease
	60+	0.3	-1.6; 2.3	Stationary	60+	-1.8*	-3.1; -0.5	Decrease
**North**					**South**			
General		2.3*	0.2; 4.5	Increase	General	-3.0*	-4.7; -1.2	Decrease
	13–19	6.2*	5.2; 7.3	Increase	13–19	-2.7*	-4.2; -1.2	Decrease
	20–24	5.7*	4.4; 7.1	Increase	20–24	-1.2	-2.9; 0.6	Stationary
	25–29	3.0*	0.7; 5.4	Increase	25–29	-3.5*	-4.6; -2.4	Decrease
	30–34	1.1	-0.9; 3.2	Stationary	30–34	-4.9*	-6; -3.7	Decrease
	35–39	0.6	-1.6; 2.9	Stationary	35–39	-4.2*	-6.4; -2	Decrease
	40–49	0.9	-1; 2.9	Stationary	40–49	-3.1*	-4.9; -1.4	Decrease
	50–59	2.2	-0.1; 4.6	Stationary	50–59	-0.6	-2.3; 1.2	Stationary
	60+	4	-0.4; 8.7	Stationary	60+	0.9	-0.8; 2.6	Stationary
**Northeast**					**Midwest**			
General		0.5	-0.8; 1.8	Stationary	General	-0.5	-1.7; 0.7	Stationary
	13–19	3.0*	1.2; 4.8	Increase	13–19	2	-9.5; 15	Stationary
	20–24	3.2*	0.9; 5.6	Increase	20–24	4.9*	2.8; 7.1	Increase
	25–29	0.5	-1.5; 2.5	Stationary	25–29	1	0; 2.1	Stationary
	30–34	-1.1	-2.6; 0.5	Stationary	30–34	-1.3*	-2.3; -0.3	Decrease
	35–39	-1.2*	-1.9; -0.5	Decrease	35–39	-2.8*	-3.6; -1.9	Decrease
	40–49	-0.4	-1.8; 1.1	Stationary	40–49	-1.5*	-2.7; -0.3	Decrease
	50–59	2.8*	1.5; 4.1	Increase	50–59	0.5	-1.2; 2.2	Stationary
	60+	4.0*	1; 7.1	Increase	60+	-0.6	-3.7; 2.6	Stationary

Statistically significant data

* p<0.05

APC: annual percent change.

Source: Research data, 2022.

In Figure 1 we show the Kernel Density Map of SEB in Brazil. We can observe that the South, Southeast, and Northeast regions exhibit large conglomerates, with SEB above 31.51 cases/10 thousand inhabitants. The lowest coefficients are distributed in the North and Midwest regions. States, such as Paraíba, Sergipe, Alagoas, Pernambuco, São Paulo, Minas Gerais, Pará, Rio Grande do Sul, and Santa Catarina, have the highest indicators.

## DISCUSSION

In this research, we identified a high number of AIDS cases in Brazil in the 16 years investigated. The highest incidence rates were respectively in the South, North, Brazil, Southeast, Midwest, and Northeast. We found that the temporal trend was decrease in Brazil and in all regions, except for the North and Northeast.

This reduction in the incidence rate represents the positivity of the work that began in the 1980s, when the first cases of AIDS were recorded in Brazil — when the National STD/AIDS Policy was created by the Brazilian Ministry of Health, and the country stood out by providing antiretroviral therapies (ART) to the entire population. The use and evolution of these drugs, in addition to reducing the transmission of the disease, reduced mortality and improved the quality of life of the affected individuals^19,20^.

It is known that underdeveloped and developing countries present significant difficulties in preventing and treating the disease, especially due to political, social, and economic issues, which are directly reflected in healthcare systems. Hence, there are enormous challenges for public health managers and professionals^3,21^, especially due to the consolidation of prevention measures, as well as the effectiveness of ART, which can improve the health and survival of individuals living with AIDS^22^.

Brazil, despite facing the aforementioned difficulties, formulated public policies from 2000 onwards with the objective of reducing inequities, which contributed to the reduction of incidence, hospitalization, and mortality indicators^23^. In addition, efforts to expand testing, treatment availability, and awareness in the Brazilian territory may also have contributed to the reduction of these indicators. In the meantime, the relevance of lower rates among young women is also highlighted, a possible reflection of health initiatives directed at women with a gynecological and obstetric focus^22^, which increases the detection rate of the disease.

In the Northern region, we found an increase in the incidence rate in the general group and in younger age groups up to 29 years. This fact may be related to the higher risk behavior of these groups, such as the increase in the number of sexual partners, the nonuse of condoms, and the increase in the number of illicit drugs^24^.

Most of the young population is composed of students, especially with a higher education level. The Centers for Disease Control and Prevention (CDC) organization states that the number of college students diagnosed with AIDS increases by 30 to 50% every year^25^. In Brazil, although there are no epidemiological data on the incidence of this condition in the university population, several studies show that these individuals are more susceptible to the disease due to risky behaviors and low knowledge of prevention measures such as condom use^26-28^ .

Furthermore, researchers highlight sexuality issues (homosexual transmission) that are identified as predictors for younger individuals, considering the lack of knowledge of important information about the disease, which results in its late diagnosis^29^. Another factor that may explain this increase concerns the lack of access to diagnostic methods or is a reflection of the inadequate control of the epidemic in the North of the country^30^. Therefore, there is an urgent need to intensify education initiatives aimed at this population, especially those who present greater risks, in addition to the indiscriminate and facilitated provision of preventive methods.

Despite this context, religious and conservative views have prevented sexual education activities in schools, which may increase the rates of unsafe sex among young people and the lack of risk perception. This represents a major challenge for public policies aimed at controlling the epidemic in Brazil. Updates to these estimates will allow professionals to monitor the progress and plan effective interventions. Moreover, depending on the granularity of the data, the model can be used to derive estimates on other subpopulations and focus interventions on the most challenging population groups at subnational levels^22^.

Overall, the more advanced age groups showed a reduction in the incidence rate, with the exception of the Northeast, where there was an increase pattern in the age groups over 50 years. Although we observed this pattern in only one region of the country, it does not differ from that found in other studies. Research conducted in China found an increase trend among men and women aged over 35 years, and the projection for the next five years (2019–2023) also showed an increase in incidence^31^.

These findings can be explained by health inequities and risky behaviors. A research conducted in South Korea, which evaluated sexual behavior and sexually transmitted infections (STIs) in older adults, identified active sexual activity with multiple partners and low adherence to condom use^32^. Another study shows that health professionals perceive older adults as asexual and that, at the level of primary health care, the request for serological tests is uncommon, especially for this population, which results in a late diagnosis of the disease, which occurs at the secondary and tertiary levels of health care^33^.

The level of education is also a point of interest when it comes to prevention. People with low level of education have limited capacity to understand knowledge of diseases and their preventive methods. This is also linked to the socioeconomic condition, which influences access to information, health, and the prevention of health issues among older adults^34^. In 2019, the Northeast had the highest illiteracy rates in Brazil, with 13.9%, according to IBGE^35^. Of these individuals, over 37% are older adults^36^. Thus, in addition to deficits in the perception of professionals in the implementation of preventive and diagnostic actions in the older adult population, limited knowledge and education, associated with social, cultural, and economic factors, may influence the outcome of the disease.

We found larger spatial conglomerates in the Southeast, South, and Northeast regions. The spatial distribution of AIDS in Brazil differs between regions. A study conducted in the state of Ceará shows that AIDS is more prevalent in cities with higher family income and, therefore, may be more associated with the risk-taking behavior of modern life than with factors related to poverty^37^.

However, given the complex spatial heterogeneity of the country, some locations may have a greater effect of socioeconomic factors on the incidence of the condition. The Northeast and the North regions have a moderate association between the occurrence of AIDS and urbanization, in addition to the correlation with inequality, vulnerability, and income^38^.

In this context, it is known that Primary Health Care (PHC) plays an extremely important role in promoting the health of the Brazilian population, in such a way that investments in this strategy for AIDS prevention are essential. Health professionals in the PHC must pay attention to this condition, which, due to treatment, has become a chronic disease, as well as to eliminate prejudices, welcome these older people, provide health education, among many actions^39^.

Studies on aggregated data are important for the epidemiological assessment of health issues, which can support the recognition of the scenario by managers and professionals, assist in decision-making and in the review of public policies and health actions, especially for the nursing team that plays crucial roles in the management, prevention, and treatment of individuals with AIDS in the primary, secondary, or tertiary care levels.

This study has limitations such as the presence of underreporting and the quality of the data used in the research. Publicly accessible secondary data suffer from loss, omission, and flaws in identifying and completing the notification form, which may underestimate or overestimate the presented information. Another limitation is the analysis time, considering that several changes can occur over a long period, such as 16 years. The temporal and spatial analysis of large regions does not allow considering the specificities of each location and time, especially in a country such as Brazil, with continental dimensions and clear differences between regions.
